# Medial temporal lobe‐cortical connectivity is lower in older adults of African ancestry with the ABCA7‐80 risk allele but not in APOE‐ε4 carriers

**DOI:** 10.1002/alz70861_108663

**Published:** 2025-12-23

**Authors:** Miray Budak, Bernadette A. Fausto, Wiktoria Piaszczynska, Payton G. White, Martina A. Ishaq, Soodeh Moallemian, Kelly N. Nudelman, Mark A. Gluck

**Affiliations:** ^1^ Rutgers University–Newark, Newark, NJ USA; ^2^ Department of Medical and Molecular Genetics, Indiana University School of Medicine, Indianapolis, IN USA

## Abstract

**Background:**

APOE‐ε4 and ABCA7‐80 (rs115550680) are two prominent genetic risk factors for Alzheimer’s disease (AD). While APOE‐ε4 is the most common genetic risk factor for AD in people of European ancestry, ABCA7‐80 is linked to late‐onset AD in individuals of African ancestry. The medial temporal lobe (MTL), an early locus of AD‐related pathology, is critical for memory and attention. Understanding genotype‐specific alterations in MTL connectivity with cortical regions is crucial for identifying early biomarkers of AD in African‐ancestry populations. To investigate MTL‐cortical connectivity in cognitively unimpaired older adults with African ancestry carrying the high‐risk ABCA7‐80 and APOE‐ε4 alleles.

**Method:**

Participants (*N* =146; Mean Age = 69.71±6.29; 110 women) were drawn from the Aging and Health Alliance study at Rutgers‐Newark. Genetic status for APOE‐ε4 and ABCA7‐80 was determined using saliva‐based genotyping. Resting‐state functional MRI (rs‐fMRI) was used to perform seed‐based connectivity analyses, focusing on MTL and hippocampal subregions (Subiculum, Cornu Ammonis 1 (CA1), Dentate Gyrus (DG)/CA3, Parahippocampal Cortex, Perirhinal Cortex, Anterolateral Entorhinal Cortex, and Posteromedial Entorhinal Cortex) and their cortical targets. Group differences in connectivity were assessed using t‐statistic maps, with age, sex, and education as covariates, and significance was determined via Threshold‐Free Cluster Enhancement (TFCE) correction (p < .05).

**Result:**

ABCA7‐80 carriers exhibited significantly reduced functional connectivity between the subiculum and the supramarginal gyrus, and between the DG/CA3 and the superior parietal lobule, compared to APOE‐ε4 carriers (TFCE corrected *p* < .05). These disruptions suggest that ABCA7‐80 carriers may exhibit early alterations in memory consolidation and attention integration networks, potentially increasing their risk of cognitive decline.

**Conclusion:**

APOE‐ε4 and ABCA7‐80 differentially shape MTL‐parietal networks in preclinical stages of AD. The observed decrease in connectivity in ABCA7‐80 carriers may reflect early dysfunction in neural networks involved in memory and attention. ABCA7‐80 may therefore serve as an early marker of neural disruption in older adults of African ancestry, providing valuable insights for genotype‐targeted interventions.

